# Reptile vector-borne diseases of zoonotic concern

**DOI:** 10.1016/j.ijppaw.2021.04.007

**Published:** 2021-04-22

**Authors:** Jairo Alfonso Mendoza-Roldan, Miguel Angel Mendoza-Roldan, Domenico Otranto

**Affiliations:** a Department of Veterinary Medicine, University of Bari, Valenzano, Italy; b Independent Veterinarian, Mar y tierra, Agost, Alicante, Spain; c Department of Pathobiology, Faculty of Veterinary Science, Bu-Ali Sina University, Hamedan, Iran

**Keywords:** Reptiles, Vectors, Mites, Ticks, Mosquitoes, Sand flies, Bacteria, *Leishmania*, *Trypanosoma*, Arboviruses, Evolution

## Abstract

Reptile vector-borne diseases (RVBDs) of zoonotic concern are caused by bacteria, protozoa and viruses transmitted by arthropod vectors, which belong to the subclass Acarina (mites and ticks) and the order Diptera (mosquitoes, sand flies and tsetse flies). The phyletic age of reptiles since their origin in the late Carboniferous, has favored vectors and pathogens to co-evolve through millions of years, bridging to the present host-vector-pathogen interactions. The origin of vector-borne diseases is dated to the early cretaceous with Trypanosomatidae species in extinct sand flies, ancestral of modern protozoan hemoparasites of zoonotic concern (e.g., *Leishmania* and *Trypanosoma*) associated to reptiles. Bacterial RVBDs are represented by microorganisms also affecting mammals of the genera *Aeromonas, Anaplasma, Borrelia*, *Coxiella*, *Ehrlichia* and *Rickettsia*, most of them having reptilian clades. Finally, reptiles may play an important role as reservoirs of arborivuses, given the low host specificity of anthropophilic mosquitoes and sand flies. In this review, vector-borne pathogens of zoonotic concern from reptiles are discussed, as well as the interactions between reptiles, arthropod vectors and the zoonotic pathogens they may transmit.

## Highlights

• Zoonotic reptile vector-borne diseases are caused by bacteria, protozoa and viruses.
• Arthropod vectors associated to reptiles belong to the subclass Acarina and order Diptera.
• Ticks and mites have been recorded molecularly positive for zoonotic bacteria.
• Sand flies most likely originated in the lower cretaceous along with trypanosomatid parasites.
• Reptiles may act as reservoirs or overwintering hosts of mosquito-borne arboviruses of zoonotic concern.

## Graphical abstract

**Figure:**
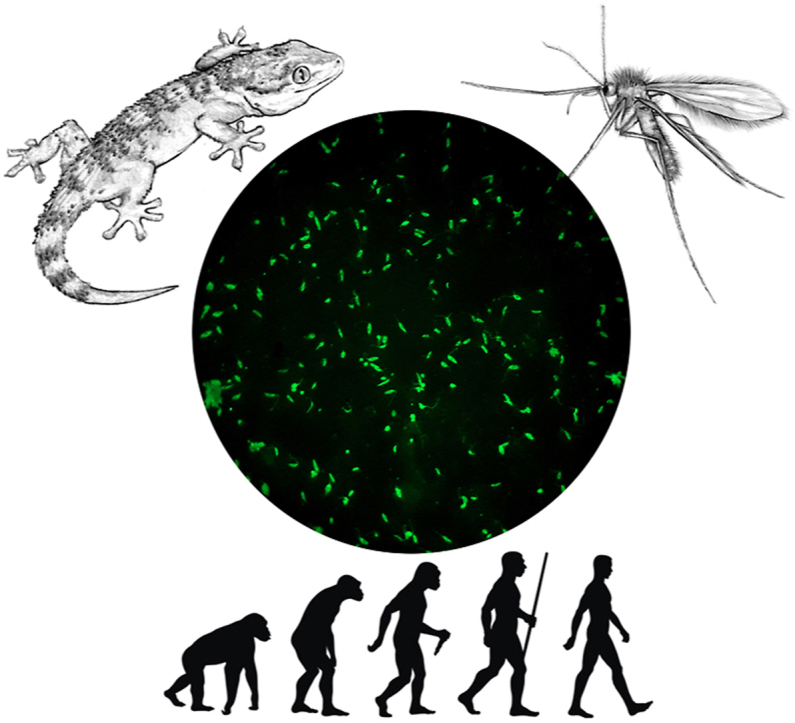

## 1 Introduction

**Fig. 1:**
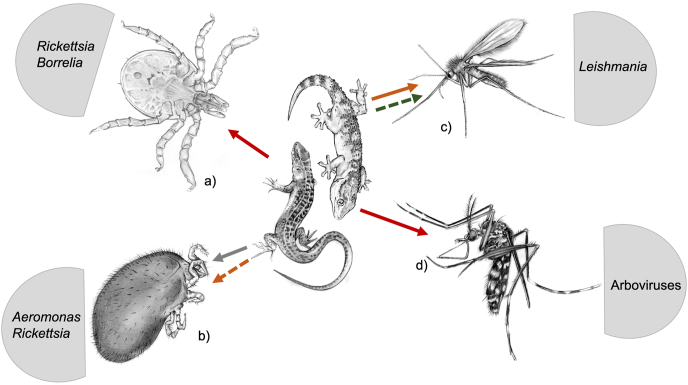
Arthropod vectors associated to reptiles represented by a *Podarcis siculus* lizard and *Tarentola mauritanica* gecko and zoonotic pathogens they may transmit. a) *Ixodes ricinus* tick larva, b) *Ophionyssus natricis* mite, c) *Sergentomyia minuta* sand fly, d) *Aedes albopictus* mosquito. Red lines represent high importance role of transmission, orange line represents medium importance role of transmission, gray line represents mechanical vector and green line represents transmission of non-pathogenic zoonotic microorganisms. Dashed lines represent neglectable knowledge on actual role of vector. (For interpretation of the references to colour in this figure legend, the reader is referred to the Web version of this article.)

Reptiles are among the most diverse and successful group of vertebrates, including more than 1200 genera and around 11,000 species (Roll et al., 2017). This class is divided in four orders: Squamata (i.e., 10,417 species of lizards, snakes, and amphisbaenians), Testudines (i.e., 351 species of turtles and tortoises), Crocodylia (i.e., 24 species of crocodiles, alligators, caimans and gavials), and Rhynchocephalia, the latter represented by a single species of living fossils named tuataras (Pincheira-Donoso et al., 2013). Since the appearance of reptiles, 310–320 million years ago in the late Carboniferous, this class of animals has scarcely changed as per their morphology, biology and ecology (Tucker and Benton, 1982; Lepetz et al., 2009). Along with them, vectors and pathogens have co-evolved through millions of years, possibly bridging to the present host-vector-pathogen interactions. Under the above circumstances, the interactions amongst reptiles, arthropod vectors and transmitted pathogens could be considered a model for unravelling the intimate relationship within the vector-borne diseases (VBDs). An example is represented by the origin of pathogenic malaria parasites, which is believed to had diverged in the half of the Eocene epoch from reptilian ancestors (Hayakawa et al., 2008). Moreover, many zoonotic diseases could have originated or are associated to a reptilian host. For example, some studies initially hypothesized that the origin of the SARS-COV-2, causative agent of the COVID-19 pandemic, were snakes (Tiwari et al., 2020; Ji et al., 2020). This is also the case of the evolution of VBDs, where many pathogens have a clade or cluster of species associated to reptiles or ectothermic tetrapods, like the reptile-associated *Borrelia* group (Morales-Diaz et al., 2020), or the reptile clade of *Leishmania* (subgenus *Sauroleishmania*) (Tuon et al., 2008). Also, some parasitic arthropods became well adapted to their reptilian host producing minimum deleterious effects on them (Bertrand et al., 2002; Bower et al., 2019), such as in the case of *Amblyomma rotundatum* ticks infesting reptiles in South America (Polo et al., 2021; Mendoza-Roldan et al., 2020a), or *Ixodes ricinus* parasitizing wild lizards (*Lacerta agilis*) in Europe (Wieczorek et al., 2020). Conversely, other parasitic arthropods (e.g., *Ophionyssus natricis* mites in snakes) may have a pronounced deleterious effect on their hosts, when there is a high parasitic load (Fuantos-Gámez et al., 2020). However, the vector-host interaction becomes noticeably important when considering vector-borne agents (i.e., bacteria, parasites, viruses) of zoonotic concern. The success of microorganisms in infecting the hosts depends on different factors acting in synergy (Prakasan et al., 2020). For example, in endemic areas of visceral leishmaniasis in Northwest China, where typical canid hosts are scarce, lizards were found to be molecularly positive for *Leishmania turanica*, *Leishmania tropica* and *Leishmania donovani* complex (Zhang et al., 2019), and snakes of *L. turanica* and *L. donovani* (Chen et al., 2019). In addition, reptiles may be infected by various zoonotic VBDs (i.e., bacterial, protozoal, viral) being the primary source of bloodmeal for arthropod vectors (i.e., ticks, mites, sand flies and mosquitoes) (Fig. 1) that equally may feed on humans (Mendoza-Roldan et al., 2020a, Mendoza-Roldan et al., 2020b, Mendoza-Roldan et al., 2021a). In this review, we discuss vector-borne pathogens associated to reptiles, as well as the interactions between reptiles, arthropod vectors and the pathogens they may transmit with a focus on those of zoonotic concern.

## 2 Arthropods feeding on reptiles

Arthropod vectors may transmit pathogens in which they partially develop (biological vectors) or are merely transported until their transmission (mechanical vectors) to a susceptible host (Di Giovanni et al., 2021). While many studies have been carried out concerning host-parasite relationship of mammals and birds with Acarina (i.e., ticks and mites) and insects (i.e., sand flies and mosquitoes), the relationships between ectoparasites and reptiles have been much less investigated (Mendoza-Roldan et al., 2020b, 2021a). In particular, knowledge on ectoparasites of reptiles mainly derive from ecological and biological studies (Mihalca, 2015), resulting in a consistent lack of information on their role as vectors of pathogens for reptiles and for mammalian species, as well as on their biological interactions and transmission modalities. Nonetheless, data on arthropod vectors of pathogens and host-arthropod association are of key importance to better understand the origin of zoonotic diseases. The relationship established by arthropods and reptiles dates back to dinosaurs when these arthropod parasites firstly appeared (Peñalver et al., 2017). For example, it is hypothesized that ticks originated in the Paleozoic Era (in the Devonian, Carboniferous or Permian periods) feeding on the ancestors of reptiles and amphibians (Dobson and Barker, 1999; Jeyaprakash and Hoy, 2009; Mans et al., 2016). While some authors indicated that ticks originated in the Mesozoic Era, between the Triassic and Jurassic periods (Balashov, 1994; Beati and Klompen, 2019), fossil data suggest that ixodid and argasid ticks already had diverged since the Cretaceous period (Poinar and Brown, 2003; Klompen and Grimaldi, 2001; Chitimia-Dobler et al., 2017; Estrada-Peña and de la Fuente, 2018).

### 2.1 Ticks and mites

On the whole, more than 500 species of mites and ticks (subclass Acarina) parasitize ectothermic tetrapods (amphibians and reptiles) worldwide (Mendoza-Roldan et al., 2020a). They belong to the orders Trombidiformes (superorder Acariformes), Mesostigmata and Ixodida (superorder Parasitiformes). In particular, the order Trombidiformes encompasses around seven families and more than 30 genera infesting reptiles and amphibians, while Mesostigmata includes five families and 18 genera developing on ectothermic tetrapod fauna (Fain, 1962).

Within Ixodida, species parasitizing reptiles and amphibians are about 100 and they belong to 8 genera within the family Ixodidae and a few Argasidae (Barros-Battesti et al., 2006, 2015; Dantas-Torres et al., 2008; Muñoz-Leal et al., 2017). Very often larval and nymphal stages feed on reptiles but may also infest mammals and birds developing in rare cases exclusively on reptiles as principal hosts (e.g., species of the genus *Amblyomma*). This is the case of *Amblyomma humerale* whose larvae and nymphs feed on mammals and reptiles, whereas adults preferentially feed on turtles and tortoises (Martins et al., 2020). The high specialization exclusively on one reptile species (monoxenous parasitism) is rare, such as in the case of *Argas* (*Microargas*) *transversus* (Argasidae) from *Chelonoidis nigra* (Hoogstraal and Kohls, 1966). The long-lasting evolution of ticks with reptiles and amphibians is also suggested by the capacity some tick species have developed to survive underwater for certain periods (Fielden et al., 2011; Giannelli et al., 2012; Bidder et al., 2019). This strategy may also be advantageous for ticks to thrive in environments that experience seasonal floods or even for those parasitizing hosts which live in close contact with the water (Luz and Faccini, 2013; Dantas-Torres et al., 2019; Kwak et al., 2021). This is the case of *A. rotundantum* parasitizing reptiles and amphibians in South America (Luz et al., 2013; Dantas-Torres et al., 2019) and of the sea snake tick *Amblyomma nitidum* that parasitizes snakes of the genus *Laticauda*, being one of the few tick species regarded as semi-marine (Kwak et al., 2021).

**Fig. 2:**
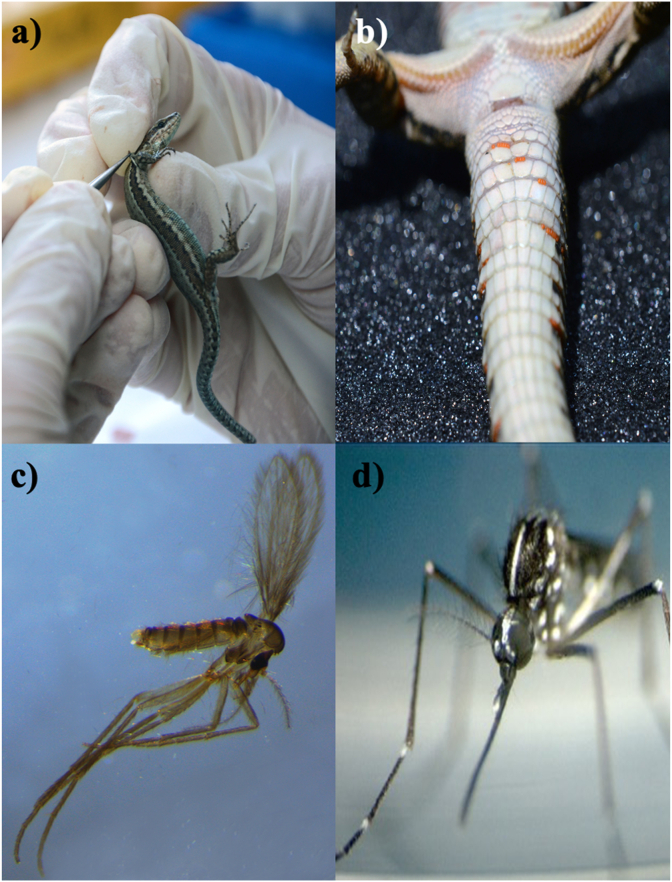
Arthropod vectors that may feed on reptiles. a) *Ixodes ricinus* larva on *Podarcis siculus* lizard being collected with tweezers, b) *Neotrombicula autumnalis* larvae mites on *Podarcis siculus* lizard, c) female *Sergentomyia minuta* phlebotomine sand fly, d) *Aedes albopictus* mosquito.

While the direct negative-effect of mites and ticks on the fitness and health status of the infested animals is overall negligible (e.g., anemia, dehydration, emaciation, dysecdysis), they may be of major importance as vectors of pathogens to other animal species including humans (Mendoza-Roldan et al., 2019, 2021b). This is the case of *I. ricinus* ticks feeding on lizards and associated to *Borrelia burgdorferi* sensu lato (Fig. 1a) (Majláthová et al., 2008; Mendoza-Roldan et al., 2019) and spotted fever group *Rickettsia* spp. (Fig. 2a) (Mendoza-Roldan et al., 2021b) (Table 1). Permanent and temporary mites and ticks may colonize different areas of the host's body with varying degrees of clinical signs. For example, most ectoparasites attach on/or inside the connective tissue underneath the scales (Mendoza-Roldan et al., 2017). Overall, preferred niches depend on the ability and size of the mite or tick, with large parasites (Ixodida and Macronyssidae) choosing areas that are unreachable after producing pruritus (e.g., head, nasal area, axillae, joints, toes and cloaca) (Chilton et al., 1992b; Bannert et al., 2000), and smaller mites (e.g., Trombiculidae, Pterygosomatidae) attaching evenly on the host body (Bertrand, 2002) or in the respiratory system of their hosts (e.g., Entonyssidae in snakes) (Fain et al., 1983). Mites parasitizing reptiles belong to the orders Trombidiformes (Acariformes) and Mesostigmata. With seven families and more than 30 genera infesting reptiles and amphibians (Zhang et al., 2011; Rezende et al., 2012), the Trombidiformes is the most represented order of mites parasitizing herpetofauna, whereas Mesostigmata includes five families and 18 genera (Lizaso, 1979, 1982). The role of mites as vectors of zoonotic pathogens has not been fully investigated although data suggest their implication as vectors for some of them, such as *Rickettsia* spp. (Fig. 2b) (Mendoza-Roldan et al., 2021a, 2021b). In spite of the paucity of information about mites, in areas where specific studies have been carried out in reptiles (e.g., in Brazil) many species have been described, as belonging to eight genera and 11 species of Trombidiformes and Mesostigmata (Mendoza-Roldan et al., 2017; Jacinavicius et al., 2018). In a comprehensive study of reptiles and amphibians in Brazil (n = 4515 specimens examined) the majority of infested animals (*n* = 170) were lizards (*n* = 72; 42.3%), infested mainly by Trombidiformes order (Trombiculidae and Pterygosomatidae) (Mendoza-Roldan et al., 2020b). Examples of mite vectors of pathogens are represented in both the Trombidiformes and Mesostigmata orders (Fig. 1b) (Table 1).

**Table 1:** Species of mites and ticks, their reptile hosts and associated zoonotic pathogens.

Type of Acarina	Species of vector	Reptile host	Country	Zoonotic pathogen	Reference
Mite	*Eutrombicula alfreddugesi*	Snakes	Brazil	*Rickettsia* sp.	Mendoza-Roldan et al. (2021a)
				*Rickettsia bellii-like*	
	*Geckobiella harrisi*	Lizards	Brazil	*Rickettsia* sp.	Mendoza-Roldan et al. (2021a)
	*Ophiogongylus rotundus*	Snakes	Brazil	*Rickettsia* sp.	Mendoza-Roldan et al. (2021a)
	*Neotrombicula autumnalis*	Lizards	Italy	*Rickettsia* sp.	Mendoza-Roldan et al. (2021b)
	*Ophionyssus natricis*	Snakes	United states	*Aeromonas hydrophila*	Camin (1984)
		Lizards	Brazil	*Rickettsia* sp.	Mendoza-Roldan et al. (2021a)
Tick	*Amblyomma chabaudi*	Tortoises	Madagascar	*Rickettsia africae*	Sanchez et al. (2019)
	*Amblyomma clypeolatum*	Tortoises	Japan	*Rickettsia* sp.	Andoh et al. (2015)
	*Amblyomma dissimile*	Freshwater turtles	Colombia	*Rickettsia* sp.	Santodomingo et al. (2018)
		Snakes			
		Lizards			
		Snakes	Mexico	*Rickettsia* sp.	Sanchez et al. (2019)
		Lizards			
			Japan	*Borrelia* sp.	Takano et al. (2010)
			Honduras	*Rickettsia* sp. strain Colombianensi	Novakova et al. (2015)
	*Amblyomma exornatum*	Monitor Lizards	Guinea Bissau	*Coxiella burnetii*	Arthur (1962)
			United Kingdom	*Ehrlichia* sp.	Mihalca (2015)
			Japan		Andoh et al. (2015)
	*Amblyomma fimbriatum*		Australia	*Rickettsia tamurae*	Sanchez et al. (2019)
	*Amblyomma flavomaculatum*		Poland	*Anaplasma phagocytophilum*	Mihalca (2015)
			Ghana	*Anaplasma* sp.	Nowak et al. (2010)
	*Amblyomma geoemydae*	Box turtles	Japan	*Rickettsia aeschlimannii*-like	Qiu et al. (2021)
				*Candidatus Ehrlichia* occidentalis	
	*Amblyomma helvolum*	Snakes	Malaysia	*Ehrlichia* sp.	Kho et al. (2015)
				Candidatus “Rickettsia johorensis	
				*A. phagocytophilum*	
	*Amblyomma latum*	Snakes	Japan	*Rickettsia* sp.	Andoh et al. (2015)
		Lizards		*Erlichia* sp.	
	*Amblyomma nitidum*	Marine snakes		*Rickettsia* sp.	Qiu et al. (2021)
				*Candidatus Ehrlichia occidentalis*	
	*Amblyomma nuttalli*	Snakes	Ghana	*C. burnetii*	Kim et al. (1978)
	*Amblyomma parvitarsum*	Lizards	Chile	*Rickettsia parkeri* strain Parvitarsum	Sanchez et al. (2019)
	*Amblyomma rotundatum*	Snakes	Brazil	*Rickettsia bellii*	Mendoza-Roldan et al. (2021a)
				*Rickettsia amblyommatis*	
	*Amblyomma sabanerae*	Freshwater turtles	United states	*Rickettsia helvetica*	Sanchez et al. (2019)
			El salvador		
	*Amblyomma sparsum*	Tortoises	Zambia	*Ehrlichia chaffeensis*	Andoh et al. (2015)
				*Candidatus* Neoehrlichia mikurensis	
				*Ehrlichia ruminantium*	Peter et al. (2002)
				*R. bellii*	Sanchez et al. (2019)
				*Rickettsia raoultii*	
			Japan	*Rickettsia* sp.	Andoh et al. (2015)
				*Erlichia* sp.	
	*Amblyomma transversale*	Snakes			
			Ghana	*Rickettsia hoogstraalii*	Sanchez et al. (2019)
	*Amblyomma trimaculatum*	Snakes	Japan	*Rickettsia* sp.	Andoh et al. (2015)
	*Amblyomma varanense*	Monitor lizards	Indonesia	*Anaplasma* sp.	Takano et al. (2019)
	*Amblyomma variegatum*	Reptiles	Congo	*C. burnetii*	Giroud (1951)
	*Bothriocroton hydrosauri*	Lizards	Australia	*Rickettsia honei*	Whiley et al. (2016)
		Snakes			
	*Bothriocroton undatum*	Monitor lizards	Australia	*Borrelia* sp.	Paneta et al. (2017)
	*Haemaphysalis sulcata*	Lizards	Italy	*R. hoogstraalii*	Sanchez et al. (2019)
	*Hyalomma aegyptium*	Tortoises	Algeria	*Rickettsia aeschlimannii*	Sanchez et al. (2019)
			Middle East	*C. burnetii*	Široký et al. (2010)
			Romania	*A. phagocytophilum*	Paș;tiu et al., 2012
				*Erlichia canis*	
				*C. burnetii*	
			Turkey	Crimean-Congo hemorrhagic fever	Kar et al. (2020)
	*Ixodes ricinus*	Lizards	Netherlands	*Rickettsia helvetica*	Sanchez et al. (2019)
		Snakes		*Rickettsia typhi*	
		Lizards	Italy	*R. helvetica*	Mendoza-Roldan et al. (2021a)
				*Rickettsia monacensis*	
			Europe	*Borrelia lusitaniae*	Mendoza-Roldan et al. (2019)
				*A. phagocytophilum*	Nieto et al. (2009)
			Italy	*Erlichia* sp	Mendoza-Roldan et al. (2019)
	*Ixodes pacificus*	Lizards	United states	*A. phagocytophilum*	Nieto et al. (2009)
				*Borrelia burgdorferi* (sensu lato)	Kuo et al. (2000)
	*Ornithodoros moubata*	Tortoises	North America	*Borrelia turicatae*	Estrada-Peña and Jongejan (1999)
	*Ornithodoros turicata*	Tortoises	Africa	*Borrelia duttoni*	
		Snakes			

Moreover, in some studies in other geographical areas (e.g., the Palearctic, Nearctic and Ethiopic regions), a high parasitic load of ticks was observed on lizards, also with no apparent negative effect on the host health (Prendeville and Hanley, 2000; Soualah-Alila et al., 2015; Dudek et al., 2016; Mendoza-Roldan et al., 2019). Indeed, lizards have been found infested by larvae and nymphs of *Ixodes pacificus* in the Nearctic region, and *I. ricinus* in the Palearctic region (Mendoza-Roldan et al., 2019). Conversely, *B. burgdorferi* sensu lato in the Neotropical region probably is maintained by birds and small mammals, rather than lizards (Barbieri et al., 2013; Ogrzewalska et al., 2016; De Oliveira et al., 2018). Furthermore, another paradigmatic example of the participation of ticks, associated to a certain level to reptiles, in the eco-epidemiology of zoonotic pathogens, is represented by *Hyalomma aegyptium*. This species of tick feeds mainly on *Testudo* tortoises in the Palearctic region, but may also feed on mammals. Given the high molecular prevalence of important zoonotic pathogens, normally associated to warm-blooded animals (i.e., *Anaplasma*, *Ehrlichia*, *Coxiella burnetii*) detected on this tick species in Romania, a possible host-switching behavior may had occurred, further increasing the zoonotic pathogens transmission implications of *H. aegyptium* (Paș;tiu et al., 2012).

### 2.2 Sand flies

Similar to other arthropod vectors mentioned above, sand flies (Diptera: Psychodidae) most likely evolved during the lower Cretaceous (105-100 mya) as they were found engorged in Burmese amber containing stages of a leishmanial trypanosomatid in their proboscis and abdominal midgut along with reptilian erythroid cells (Poinar and Poinar, 2004b). Incidentally, the fossil sand fly morphologically resembled those of the genus *Sergentomyia*, which includes species feeding on ectothermic animals (Fig. 1c) (Alkan et al., 2013). Based on these results, authors hypothetized that the extinction of dinosaurs could have been caused by epidemics of *Leishmania* spp. (Desowitz, 1991). The evolutionary history of Phlebotominae sand flies is directly linked to hemoparasites rather than to their definitive hosts. Indeed, sand fly species are distributed worldwide (Torres-Guerrero et al., 2017), mainly in the tropical and neotropical regions (Lozano-Sardaneta et al., 2018), and feed on diverse species of vertebrates. Consequently, sand flies are opportunistic blood feeders, depending on host availability rather than specific attractiveness (Pérez-Cutillas et al., 2020; Cotteaux-Lautard et al., 2016). For example, *Lutzomyia* (*Helcocyrtomyia*) *apache*, considered as an exclusive feeder of warm-blood vertebrates, may also feed on the western fence lizards (*Sceloporus occidentalis*; Reeves, 2009). The same occurred for *Sergentomyia minuta* (Fig. 2c), which may feed on lizards as well as on mammals (Bravo-Barriga et al., 2015; González et al., 2020). Therefore, the role of Squamata reptile populations and the ubiquitous distribution of sand fly species is of critical importance to understand the epidemiology of trypanosomid flagellates in endemic regions. Phlebotomine sand flies are the single natural vector of *Leishmania* spp. and may also be involved in the transmission of Arboviruses (Phlebovirus) and *Bartonella* sp. to humans (Ready, 2013). The protozoan *Leishmania* has highjacked the predatory mechanisms of the sand fly, enabling it to feed on potential hosts as it remains insatiate (Akhoundi et al., 2016). *Leishmania* spp. ancestors were divided in *Sauroleishmania* and current *Leishmania* genus (Killick-Kendrick et al., 1986). Nevertheless, the establishment of the sustained cycle between vector and vertebrate species, probably occurred during the Paleocene, after the appearance of placental mammals (Bates, 2007) (Table 2).

**Table 2:** Sand fly species, their reptilian blood meal source and associated zoonotic pathogens.

Phlebotomine species	Host species	Country	Zoonotic pathogen	Reference
*Phlebotomus chinensis*	Lizards	China		Zhang et al. (2019)
*Phlebotomus longiductus*				
*Phlebotomus wui*			*Leishmaniatropica*	
*Phlebotomus alexandri*			*Leishmania donovani*	
*Phlebotomus clydei*	Lizards	Kenya	*Leishmania adleri*	Heisch (1958)
*Phlebotomus kazerun*	Lizards	Pakistan	*Trypanosoma* sp.	Kato et al. (2010)
*Phebotomus perniciosus*		France	Toscana Virus	Cotteaux-Lautard et al. (2016)
*Sergentomyia minuta*		Spain	*Leishmania* sp.	Gonzalez et al. (2019)
		Spain	*Leishmania tarentolae*	Bravo-Barriga et al. (2015)
	Humans	Italy	*Leishmania donovani* complex	Abbate et al. (2020)
			*L* . *tarentolae*	
		France	Toscana Virus	Charrel et al. (2006)
*Sergentomyia* (*Sergentomyia*) *dentata*	Lizards	Iran	*L. adleri*	Maleki-Ravasan et al. (2008)
*Sergentomyia* sp.	Lizards	Worldwide	*Sauroleishmania* spp.	Lozano-Sardaneta et al. (2018)

### 2.3 Mosquitoes

Mosquitoes (Diptera, Culicidae) are well known vectors of zoonotic pathogens (Fig. 1d), such as viruses causing diseases (e.g., Dengue fever, Yellow fever, West Nile Virus, Equine Encephalitis, Zika) or protozoa causing malaria (Table 3) (Chiang and Reeves, 1962; Benelli and Mehlhorn, 2016). The feeding patterns and host preferences may be considered diverse and overlapped, therefore, intraspecific and interspecific (e.g., mammalian, avian, reptilian hosts) transmission of pathogens is likely in some regions, depending on the availability or selection of the definitive host (Shahhosseini et al., 2018). The role of reptiles in the maintenance of mosquito-borne diseases is due to the noteworthy fragment of Chordata biomass they constitute in the terrestrial biosphere and to their potential role as reservoirs of zoonotic diseases. For example, *Culex* spp. feed on reptilian populations in Southern United States of America, both as generalist or specialized feeders, and they may harbor arboviruses (Burkett-Cadena et al., 2008). Similarly, anophelines are indiscriminate blood feeders of mammals, birds, reptiles and humans, a behavior that is determined by the potential host abundance, their anthropophilic attitude (Bashar et al., 2012), searching patterns, access to hosts and environmental characteristics (Silva-Santos et al., 2019). For instance, host richness and habitat have a bold effect on the distribution and abundance of *Culex peccator* and *Culex territans* mosquitoes, which are blood feeders of reptiles and amphibians (Burkett-Cadena et al., 2013). Reports of mosquito-borne pathogens in reptile populations around the world raise concern on the potential role as reservoirs or overwintering hosts in both endemic and exotic regions (Table 3).

**Table 3:** Mosquito species, their reptilian blood meal source and associated zoonotic viral disease.

Mosquito species	Host	Country	Disease	Reference
*Aedes albopictus*	Squamata reptiles	Cuba	Zika virus	Gutiérrez-Bugallo et al. (2019)
*Aedes aegypti*				
*Aedes notoscriptus*				
*Aedes vexans*				
*Ae. Vittatus*				
*Ae. luteocephalus*				
*Aedes (Och.) camptorhynchus*				
*Culiseta melanura*	Lizards	USA	Eastern equine encephalitis virus (EEEV)	Graham et al. (2012)
	Snakes			
	Turtles			
Inoculation (lab conditions)	Crodociles	USA	Chikungunya virus	Bosco-Lauth et al. (2018)
	Lizards			
	Snakes			
	Turtles			
*Culex tarsalis*	Snakes	USA	Western equine encephalitis (WEE)	Thomas and Eklund (1962)
*Culex* sp.	Alligators	Israel	West Nile Virus	Steinman et al. (2003)
	Monitor lizards			
	Crocodiles			
In vitro	Lizards	Germany	Rift Valley fever phlebovirus (RVFV)	Rissmann et al. (2020)

## 3 Zoonotic vector-borne pathogens associated to reptiles

Reptiles may harbor a myriad of organisms, such as parasites, bacteria, fungi, protozoa and viruses, many being innocuous to them. Hence, reptiles may act as hosts of zoonotic pathogens associated to Acarina subclass (i.e., mites and ticks) or Diptera (i.e., mosquitoes and sand flies) (Václav et al., 2011; Ebani, 2017; Mendoza-Roldan et al., 2020b). Those microorganisms that cause RVBDs can be separated accordingly, being bacteria mainly associated to Acarina, viruses to Diptera and protozoa to both groups of vectors (Mendoza-Roldan et al., 2021a).

### 3.1 Bacteria

Within the vector-borne bacteria that are, in certain way, associated to reptiles, those of zoonotic importance belong to the genera *Aeromonas, Anaplasma, Borrelia*, *Coxiella*, *Ehrlichia* and *Rickettsia* (Table 1). In addition, there is a single report of *Bartonella henselae* or a species genetically related to *Bartonella vinsonii* subsp. *berkhoffii* in marine turtles (Valentine et al., 2007).

#### 3.1.1 Aeromonas

This genus of bacteria is an important pathogen for reptiles and transmission to humans is mainly water-borne (i.e., through contact of wounds and/or ingestion with contaminated water or reptile meat, and wounds produced by reptiles in contact or living in contaminated water) (Lupescu and Baraitareanu, 2015; Ebani et al., 2008; Miranda et al., 2017). However, macronyssid mites *O. natricis* (Fig. 1b) can be mechanical vectors of *Aeromonas hydrophila*, mainly in snakes (Camin, 1984; Jacobson et al., 2007; Lupescu and Baraitareanu, 2015). Reptiles develop systemic disease due to *Aeromonas* spp., therefore they are not effective reservoirs for these bacteria. Generally, infection occurs after mechanic transmission events such as, trauma, secondary infection of abscesses, mite infestation or stress due to suboptimal environmental conditions (Lupescu and Baraitareanu, 2015; Thomas et al., 2020). Infection in reptiles may induce systemic disease (i.e., stomatitis, sepsis, pneumonia) or be asymptomatic, acting *A. hydrophila* as an opportunistic pathogen (Jacobson et al., 2007). Zoonotic vector-borne risk of infection of *A. hydrophila* from reptiles is given from previous reports of *O*. *natricis* mites infesting humans (Schultz, 1975; Amanatfard et al., 2014), causing gastrointestinal symptoms, such as diarrhea, emesis and abdominal pain (Lupescu and Baraitareanu, 2015).

#### 3.1.2 *Anaplasma* and *Ehrlichia*

The genus *Anaplasma* comprises species of pathogenic bacteria mainly transmitted by ticks. These Gram-negative bacteria replicate in vertebrate and invertebrate hosts, and can cause severe symptoms and even death in animals, including humans (Crosby et al., 2021). Among these potentially fatal bacteria, the most important is *Anaplasma phagocytophilum*, the causative agent of granulocytic anaplasmosis (GA) (Nieto et al., 2009). Although main vectors of this pathogen (i.e., *I. pacificus* in the Nearctic and *I. ricinus* in the Palearctic) occasionally feed on reptiles, especially in their immature stages (i.e., larvae and nymphs), studies have shown that reptiles (i.e., lizards and snakes) play a minor role as reservoirs of GA (Nieto et al., 2009). In addition, *Anaplasma* spp. have been molecularly identified in tick species associated to reptiles to a certain level (e.g., *I. ricinus* in central and western Europe and *H. aegyptium* in eastern Europe; Václav et al., 2011; Tijsse-Klasen et al., 2010; Paștiu et al., 2012). Other tick species strictly associated with reptiles such as *Amblyomma flavomaculatum* (known as yellow-spotted monitor lizard tick from Ghana) and *Amblyomma varanense* (the Asian monitor lizard tick from Indonesia) were also detected positive for *Anaplasma* spp. (Nowak et al., 2010; Takano et al., 2019). These *Anaplasma* spp. were genetically similar to species affecting cattle (e.g., *Anaplasma marginale* and *Anaplasma bovis*), or *A. phagocytophilum*. Considering that reptiles are widely traded in the international pet market, it is pivotal to monitor imported animals to avoid the spreading of these pathogens and their vectors (Mihalca, 2015; Bezerra-Santos et al., 2021a, Bezerra-Santos et al., 2021b).

Recently, other groups of *Anaplasmataceae* have been detected from reptiles or their ectoparasites, such as *Candidatus* Anaplasma testudines detected in *Gopherus polyphemus* tortoises in Florida, United States (Crosby et al., 2021). In addition, *Canditatus* Cryptoplasma sp. REP was described from *Lacerta viridis* lizards and *I. ricinus* ticks in Slovakia (Kočíková et al., 2018), and *Podarcis* spp. and *I. ricinus* ticks from Italy (Mendoza-Roldan et al., 2021b). Both of these species of bacteria have an unknow pathogenicity, yet *Candidatus* Anaplasma testudines seems to be pathogenic to its natural reservoir. In addition, *Ehrlichia* spp. have been detected in different Acarina ectoparasites of reptiles worldwide. *Ehrlichia ruminantium*, the causative agent of heartwater disease, common to ruminants and that can occasionally infect humans, has been reported in *Amblyomma sparsum* from leopard tortoises imported into the United States from Zambia (Peter et al., 2002; Omondi et al., 2017), *Ehrlichia chaffeensis* and *Candidatus* Neoehrlichia mikurensis were detected in *Amblyomma* spp. from reptiles imported to Japan (Andoh et al., 2015). Possible new species of *Ehrlichia* were detected in *Amblyomma* spp. from sea snakes and tortoises also from Japan, closely related to *Candidatus* Ehrlichia occidentalis. Recent studies highlighted that the diversity of ehrlichial agents might be underestimated and the pathogenicity remains still unknown (Qiu et al., 2021). Other ehrlichial agents were detected from *H. aegyptium* ticks from Palearctic tortoises in Romania, *I. ricinus* ticks from lizards of Italy and *Amblyomma* spp. from snakes of Malaysia (Paș;tiu et al., 2012; Kho et al., 2015; Mendoza-Roldan et al., 2021b), which further indicates that the diversity of ehrlichial microorganisms infecting reptiles is presently underestimated in their pathogenicity, distribution and evolution.

#### 3.1.3 Borrelia

*Borrelia* are spirochete bacteria divided in the relapsing fever, the reptilian *Borrelia*, monotreme associated *Borrelia*, and the Lyme borreliosis groups. This latter group englobes around 20 species within the *B. burgdorferi* sensu lato complex, nine of which can be pathogenic to animals and humans (Majláthová et al., 2008; Mendoza-Roldan et al., 2019). Lyme disease and other borrelioses include species such as *Borrelia lusitaniae*, a species pathogenic to humans, that has reptiles as natural reservoirs. Ticks of the genus *Ixodes* (e.g., *I. ricinus, I. pacificus*, *Ixodes persulcatus* and *Ixodes scapularis*) are vectors of these bacteria (Kuo et al., 2000; Szekeres et al., 2016; MacDonald et al., 2017; Mendoza-Roldan et al., 2019). Moreover, Lyme disease species are likely associated to lacertid lizards, being natural reservoirs (Majláthová et al., 2006, 2008; Mendoza-Roldan et al., 2019), or refractory to the infection (e.g., species of lizards in the United States) by means of complement-mediated killing effect (Kuo et al., 2000). Similarly, some species of lacertid lizards seem to be incompetent hosts for many pathogenic *Borrelia* spp. in Europe (i.e., *Lacerta* spp.), due to borrelicidal effect of blood components that can reduce the bacterial load in infected ticks. Thus, some species of lizards, in specific epidemiological contexts, might reduce the prevalence of borrelial bacteria resulting in a zooprophylactic effect or reducing the vectors that can feed on competent hosts (Tijsse-Klasen et al., 2010). Additionally, a separate clade of reptile-associated *Borrelia*, with no demonstrated pathogenicity, has been detected in Turkey, Mexico, Japan, and Australia from reptiles (i.e., varanid lizards, snakes and tortoises) and ticks (i.e., *Amblyomma* spp., *Bothriocroton* spp., *H. aegyptium*) (Güner et al., 2003; Takano et al., 2010; Panetta et al., 2017; Morales-Diaz et al., 2020; Colunga-Salas et al., 2020). This group of borrelial agents, and also those from the relapsing fever group, have been detected in imported reptiles to non-endemic areas together with their ticks, highlighting the need of quarantine and control measures (Takano et al., 2010; Colunga-Salas et al., 2020). The origin of these distinct groups of *Borrelia* is still not clear, though phylogenetic analyses showed that the reptilian *Borrelia* spp. diverged from a common ancestor of relapsing fever *Borrelia* (Takano et al., 2010). Conversely, main clades of *Borrelia* (i.e., Lyme disease and relapsing fever) are thought to have co-evolved when Ixodidae and Argasidae ticks diverged. Given that reptile-*Borrelia* group is associated with ixodid ticks, current hypothesis suggest that a switching event could have occurred, either by host or vector switching (Charleston and Perkins, 2003). In addition, since ticks from both families may occur in sympatry on the same species of reptile host, it is likely that co-feeding and vector-switching events could have happened in the past, thus originating this reptile-associated monophyletic group.

#### 3.1.4 Coxiella

*Coxiella* is a genus of obligatory intracellular Gram-negative bacteria, with only one species described (i.e., *Coxiella burnetii*), the causative agent of zoonotic Q fever (Johnson-Delaney, 1996; Široký et al., 2010). Reptiles and their ticks can act as reservoirs, as for example *H. aegyptium* tick which parasitizes Mediterranean chelonians (Široký et al., 2010; Paș;tiu et al., 2012). Other reptilian ticks have been recorded as vectors of *C. burnetii*, such as *Amblyomma exornatum* from Guinea Bissau (Arthur, 1962) and *Amblyomma variegatum* in Africa (Giroud, 1951). Importantly, an outbreak of Q fever was described in New York, USA in people that had contact with imported *Python regius* snakes parasitized with *Amblyomma nuttalli* from Ghana (Kim et al., 1978). Nonetheless, *Coxiella* has been found to be a common symbiont of ticks (Machado-Ferreira et al., 2016; Špitalská et al., 2018).

#### 3.1.5 Rickettsia

*Rickettsia* are Gram-negative, aerobic and obligate intracellular bacteria which multiply by binary fission and are associated with invertebrate vectors (Parola et al., 2005). As mentioned before, reptiles participate directly in the epidemiology of some pathogens of both the Rickettsiales order and the Rickettsiaceae family (Andoh et al., 2015; Novakova et al., 2015). A representative species of *Rickettsia* of the ancestral group, commonly associated to ticks of ectothermic tetrapods in the Americas, is *Rickettsia bellii* (Barbieri et al., 2012; Andoh et al., 2015; Ogrzewalska et al., 2019; Mendoza-Roldan et al., 2021a). This basal clade, seems to have originated from herbivorous arthropods or non-blood feeding hosts, suggesting a horizontal transmission. Indeed, the *R. bellii* clade is currently linked to arthropod vectors (i.e., ticks) and rarely or unlikely infects vertebrate hosts, thus, demonstrating the cryptic position of this group, and that the vector capacity originated in the transitional group of *Rickettsia* (e.g., *Rickettsia akari* and *Rickettsia australis*) (Weinert et al., 2009). While the pathogenicity of *R. bellii* to vertebrate hosts is still unknow, most of the *Rickettsia* species of zoonotic concern, associated to reptiles, are englobed in the Spotted Fever Group (SFG). For example, *Rickettsia honei*, the causative agent of Flinders Island spotted fever, was first described from *Bothriocroton hydrosauri* from lizards and snakes (Stenos et al., 2003; Whiley et al., 2016). Other eight species of SFG *Rickettsia* have been detected in ectoparasites and in reptiles, such as a rickettsial disease in humans, known as African Fever, caused by *Rickettsia africae* and transmitted by *A. variegatum* (Parola et al., 1999). This rickettsial disease has been detected in ticks infesting reptiles imported into North America (Burridge and Simmons, 2003). Moreover, a species similar to *Rickettsia anan* was detected in *A. exornatum* ticks in varanid lizards imported to the USA (Reeves, 2006). In Europe, SFG *Rickettsia* are represented in reptiles by species such as *Rickettsia helvetica* and *Rickettsia monacensis* detected in ticks, such as *I. ricinus* (Fig. 1a) and in blood and tail of lacertid lizards (Mendoza-Roldan et al., 2021b). Other rickettsial species reported in ticks, and in some cases mites, from reptiles are *Rickettsia aeschlimannii*, *Rickettsia amblyommatis, Rickettsia hoogstraalii, Rickettsia massiliae*, *Rickettsia raoultii*, *Rickettsia rhipicephali*, *Rickettsia tamurae* and *Rickettsia typhi* (Sánchez-Montes et al., 2019). Genera of ticks that have been found infected with *Rickettsia* spp. are *Amblyomma*, *Bothriocroton*, *Dermacentor*, *Haemaphysalis*, *Hyalomma*, and *Ixodes*. On the other hand, mite species recorded positive to *Rickettsia* spp. belong to the families Ixodorhynchidae, Macronyssidae, Pterygosomatidae and Trombiculidae (Sánchez-Montes et al., 2019; Mendoza-Roldan et al., 2021a). Molecular diagnosis of *Rickettsia* spp. in reptile tissues has been achieved only in Europe in lacertid lizards from the genus *Lacerta* (e.g., *L. agilis* and *L. viridis*) and *Podarcis* (e.g., *Podarcis muralis* and *Podarcis siculus*) (Sánchez-Montes et al., 2019; Mendoza-Roldan et al., 2021b). An important role of reptiles in the epidemiology of rickettsial agents is given by the international reptile trade, where reptiles are imported with their ectoparasites harboring *Rickettsia* spp. (Burridge and Simmons, 2003; Pietzsch et al., 2006; Mihalca, 2015; Barradas et al., 2020; Bezerra-Santos et al., 2021a, 2021b). In fact, given that some tick species that usually parasitize reptiles can also infest humans, the risk of emergence of rickettsial agents in non-endemic areas exists (Norval et al., 2020).

### 3.2 Protozoa

Vector-borne protozoa associated to reptiles are represented by hemoparasites (i.e., plasmodiids, hemogregarines, and trypanosomatid flagellates), which have a greater diversity than those of mammals and birds. The higher diversity in species associated to reptiles could be due to their isolation and the ancestral features of ectothermic tetrapods (Telford, 2009). Nonetheless, those of zoonotic concern associated to reptiles belong solely to the family Trypanosomatidae (Poinar and Poinar, 2004a). Importantly, *Trypanosoma brucei*, the causative agent of sleeping sickness, was detected in monitor lizards from Kenya (Njagu et al., 1999). Incidentally, this group of lizards has been pointed out as wild hosts for the tsetse fly (*Glossina fuscipes fuscipes*) in Uganda (Waiswa et al., 2003). Accordingly, experimental evidence suggests that reptiles could be potential reservoirs of this protozoa (Woo et al., 1969). Furthermore, reptile associated *Trypanosoma* spp., especially from snakes, may be vectored by sand flies (Viola et al., 2008). Also, studies indicate that vector-borne Trypanosomatidae represented in the genus *Paleoleishmania* originated in the early Cretaceous. This genus was found in sand flies from Cretaceous Burmese amber (Poinar and Poinar, 2004a). Other *Paleoleishmania* species were described from extinct species of sand flies (i.e., *Lutzomyia adiketis*) from Dominican amber (Poinar, 2008). In addition, *Palaeomyia burmitis* was also identified with different stages of a leishmanial trypanosomatid, which had nucleated blood cells of reptilian origin (Poinar and Poinar, 2004b). Despite evidence of *Leishmania* divergence in the Cretaceous, it is still not clear whether this genus originated from the New or Old World, yet, most likely trypanosomatids may have originated in different localities and at different time points over the past 100 million years (Poinar, 2008). More importantly, different hypothesis suggest that *Leishmania* spp. spread through the forming continents following the migration of vectors and their hosts. Also, it is hypothesized that definitive hosts of primitive *Leishmania* most likely were reptiles or primitive mammals (Tuon et al., 2008). The species of *Leishmania* that infect reptiles belong to the subclade *Sauroleishmania*, which is a sister group of the pathogenic species of mammalian *Leishmania*, with around 10 species infecting reptiles (Ovezmukhammedov, 1991). Phylogenetic inference supports the origin of lizard *Leishmania* from parasites of mammals (Klatt et al., 2019). Thus, species of *Leishmania* typical of reptiles could transiently infect mammals and vice versa. For example, *Leishmania adleri* from lacertid lizards may produce cutaneous leishmaniasis in mammals (Manson-Bahr and Heisch, 1961; Coughlan et al., 2017). Also, *Leishmania tarentolae* from geckoes has been molecularly detected in human mummies from Brazil (Novo et al., 2015), and human blood from Italy (Pombi et al., 2020). Additionally, *S. minuta* (Fig. 1c), the putative vector of this *Leishmania* sp., has been recently detected feeding from humans, also in Italy (Table 2) (Abbate et al., 2020). The role of *L. tarentolae* infection in protecting mammals against other pathogenic *Leishmania* spp. needs to be further investigated also considering the promising results of preliminary heterologous vaccination attempts (Klatt et al., 2019). On the other hand, reptiles could also act as reservoirs of pathogenic *Leishmania* spp. in areas where primary hosts do not occur or where reptiles and typical hosts live in sympatry. Recent studies have detected pathogenic *Leishmania*, such as *L. tropica*, *L. donovani* and *L. turanica* in lizards and snakes in northwestern China (Zhang et al., 2019; Chen et al., 2019). Given all of the above, future studies should focus on the role reptiles could have in the epidemiology of leishmaniasis and trypanosomiasis.

### 3.3 Viruses

Reptiles and amphibians may have an important role as reservoirs or overwintering hosts for viruses, mainly arboviruses. Many species of mosquitoes may feed on reptiles, including medically important anthropophilic species such as *Aedes aegypti* and *Aedes albopictus* (Fig. 1d; 2d) (Bosco-Lauth et al., 2018). In addition, most groups of reptiles (i.e., Testudines, Squamata, Crocodylia) have been found serologically and molecularly positive for various arboviruses (Steinman et al., 2003). In fact, many reptile species are considered reservoirs for other arboviruses such as western and eastern equine encephalites, Venezuelan equine encephalitis, West Nile Virus, and most recently Chikungunya virus (Burton et al., 1966; Bingham et al., 2012; Bosco-Lauth et al., 2018). Moreover, given the convergent evolution of hematophagous Diptera and terrestrial vertebrates, blood meal identification has proven that arbovirus vectors may predominantly feed on reptiles (Cupp et al., 2004; Burkett-Cadena et al., 2008). Importantly, *Culex tarsalis* mosquitoes may feed on reptiles such as the garter snake, that can maintain the virus of the western equine encephalitis during winter, and then infect other hosts. Thus, snakes maintain the virus during brumation (overwintering). Other viruses that are related to reptiles are the Japanese encephalitis and Zika viruses (Thomas and Eklund, 1962; Oya et al., 1983; Bueno et al., 2016). Furthermore, reptiles could be involved to a lesser extent in the maintenance of Rift Valley fever phlebovirus (Rissmann et al., 2020). Other phleboviruses have been identified in the herpetophilic sand fly *S. minuta* in France, such as the Toscana virus (Table 3) (Charrel et al., 2006).

Finally, *Testudo* tortoises may serve as primary hosts of *H. aegyptium* ticks, that have been found as competent vectors of Crimean-Congo hemorrhagic fever (CCHF). This disease is caused by a zoonotic *Bunyavirales* that is distributed through Africa, the Balkans, the Middle East, and Western Asia (Kar et al., 2020). While the primary transmission cycle of CCHF is guaranteed by birds, mammals and associated *Hyalomma marginatum* ticks in the western Palearctic, tortoises, along with *H. aegyptium* tick vectors, play a role in the cryptic transmission cycle (Široký et al., 2014; Kar et al., 2020).

## 4 Conclusions

Studying RVBDs of zoonotic concern may aid to elucidate the origins of modern VBDs (i.e., bacteria, protozoa, viruses). Arthropod vectors associated to reptiles belong to two groups, Acarina subclade (i.e., mites and ticks) and Diptera order (i.e., mosquitoes, sand flies and tsetse flies). The evolution of the hematophagous behavior of these invertebrates is strictly linked to ectothermic tetrapods whereas the origin of VBDs may be dated back to the early Cretaceous, at least for protozoan parasites. Bacterial RVBDs are represented by genera that commonly affect also mammals (e.g., *Aeromonas, Anaplasma, Borrelia*, *Coxiella*, *Ehrlichia* and *Rickettsia*), most of which have a clade associated to reptiles. Protozoan hemoparasites of reptiles of zoonotic concern belong to the family Trypanosomatidae and their origin is related to reptiles and other cretaceous creatures with nucleated erythrocytes. Although some zoonotic species of *Leishmania* and *Trypanosoma* may infect reptiles, their role as reservoirs and hosts has not been fully elucidated. On the other hand, reptiles may be of relevance as primary hosts of viruses, especially arboviruses, or for their maintenance (e.g., overwintering), given the low host specificity of anthropophilic mosquitoes and sand flies.

Moreover, the COVID-19 pandemic has highlighted the role that wildlife can have in the emergence of new zoonotic diseases given the anthropic pressure on forested populations of animals, including reptiles. Certainly, future studies on RVBDs are advocated to reveal the role of reptiles in different epidemiological contexts and geographical areas, thus reducing the risk of zoonotic transmission through proper control and preventative measures.

## Declaration of interests

We undersigned Authors of the manuscript entitled “Reptile vector-borne diseases and the origin of zoonoses” declare to have no any competing interests.

## References

[bib1] Abbate J.M., Maia C., Pereira A., Arfuso F., Gaglio G., Rizzo M., Brianti E. (2020). Identification of trypanosomatids and blood feeding preferences of phlebotomine sand fly species common in Sicily. Southern Italy. PloS one.

[bib2] Akhoundi M., Kuhls K., Cannet A., Votýpka J., Marty P., Delaunay P., Sereno D. (2016). A Historical overview of the classification, evolution, and dispersion of *Leishmania* parasites and sandflies. PLoS Neglected Trop. Dis..

[bib3] Alkan C., Bichaud L., de Lamballerie X., Alten B., Gould E.A., Charrel R.N. (2013). Sandfly-borne phleboviruses of Eurasia and Africa: epidemiology, genetic diversity, geographic range, control measures. Antivir. Res..

[bib4] Amanatfard E., Youssefi M.R., Barimani A. (2014). Human dermatitis caused by *Ophionyssus natricis*, a snake mite. Iran. J. Parasitol..

[bib5] Andoh M., Sakata A., Takano A., Kawabata H., Fujita H., Une Y., Ando S. (2015). Detection of *Rickettsia* and *Ehrlichia* spp. in ticks associated with exotic reptiles and amphibians imported into Japan. PloS One.

[bib6] Arthur D.R. (1962). Ticks and Disease. *Ticks And Disease*.

[bib7] Balashov Y. (1994). Importance of continental drift in the distribution an evolution of ixodid ticks. Entomol. Rev..

[bib8] Bannert B., Karaca H.Y., Wohltmann A. (2000). Life cycle and parasitic interaction of the lizard-parasitizing mite *Ophionyssus galloticolus* (Acari: gamasida: Macronyssidae), with remarks about the evolutionary consequences of parasitism in mites. Exp. Appl. Acarol..

[bib9] Barbieri A.R., Romero L., Labruna M.B. (2012). *Rickettsia bellii* infecting *Amblyomma sabanerae* ticks in El Salvador. Pathog. Glob. Health.

[bib10] Barbieri A.M., Venzal J.M., Marcili A., Almeida A.P., González E.M., Labruna M.B. (2013). *Borrelia burgdorferi* sensu lato infecting ticks of the *Ixodes ricinus* complex in Uruguay: first report for the Southern Hemisphere. Vector Borne Zoonotic Dis..

[bib11] Barradas P.F., Mesquita J.R., Lima C., Cardoso L., Alho A.M., Ferreira P., Gärtner F. (2020). Pathogenic *Rickettsia* in ticks of spur‐thighed tortoise (*Testudo graeca*) sold in a Qatar live animal market. Transbound Emerg Dis.

[bib12] Barros-Battesti D.M., Arzua M., Bechara G.H. (2006). Carrapatos de importância médico-veterinária da região neotropical: um guia ilustrado para identificação de espécies. Carrapatos de importância médico-veterinária da região neotropical: um guia ilustrado para identificação de espécies. xvi-223.

[bib13] Barros-Battesti D.M., Landulfo G.A., Luz H.R., Marcili A., Onofrio V.C., Famadas K.M. (2015). Ornithodoros faccinii n. sp. (Acari: Ixodida: Argasidae) parasitizing the frog *Thoropa miliaris* (Amphibia: Anura: cycloramphidae) in Brazil. Parasites Vectors.

[bib14] Bashar K., Tuno N., Ahmed T.U., Howlader A.J. (2012). Blood-feeding patterns of *Anopheles* mosquitoes in a malaria-endemic area of Bangladesh. Parasites Vectors.

[bib15] Bates P.A. (2007). Transmission of *Leishmania* metacyclic promastigotes by phlebotomine sand flies. Int. J. Parasitol..

[bib16] Beati L., Klompen H. (2019). Phylogeography of ticks (Acari: Ixodida). Annu. Rev. Entomol..

[bib17] Benelli G., Mehlhorn H. (2016). Declining malaria, rising of dengue and Zika virus: insights for mosquito vector control. Parasitol. Res..

[bib18] Bertrand M. (2002). Morphologic Adaptations to Parasitism on Reptiles: Pterygosomatidae (Prostigmata: Raphignathina). Acarid Phylogeny and Evolution: Adaptation in Mites and Ticks.

[bib19] Bezerra-Santos M.A., Mendoza-Roldan J.A., Thompson R., Dantas-Torres F., Otranto D. (2021). Illegal wildlife trade: a gateway to zoonotic infectious diseases. Trends Parasitol..

[bib20] Bezerra-Santos M.A., Mendoza-Roldan J.A., Thompson R.C.A., Dantas-Torres F., Otranto D. (2021). Legal versus illegal wildlife trade: zoonotic disease risks. Trends Parasitol..

[bib21] Bidder L.A., Asmussen K.M., Campbell S.E., Goffigan K.A., Gaff H.D. (2019). Assessing the underwater survival of two tick species, *Amblyomma americanum* and *Amblyomma maculatum*. Ticks Tick Borne Dis.

[bib22] Bingham A.M., Graham S.P., Burkett-Cadena N.D., White G.S., Hassan H.K., Unnasch T.R. (2012). Detection of eastern equine encephalomyelitis virus RNA in North American snakes. Am. J. Trop. Med. Hyg..

[bib23] Bosco-Lauth A.M., Hartwig A.E., Bowen R.A. (2018). Reptiles and amphibians as potential reservoir hosts of Chikungunya virus. Am. J. Trop. Med. Hyg..

[bib24] Bower D.S., Brannelly L.A., McDonald C.A., Webb R.J., Greenspan S.E., Vickers M., Gardner M.G., Greenlees M.J. (2019). A review of the role of parasites in the ecology of reptiles and amphibians. Austral Ecol..

[bib25] Bueno M.G., Martinez N., Abdalla L., Duarte dos Santos C.N., Chame M. (2016). Animals in the Zika virus life cycle: what to expect from megadiverse Latin American countries. PLoS Neglected Trop. Dis..

[bib26] Burton A.N., McLintock J., Rempel J.G. (1966). Western equine encephalitis virus in Saskatchewan garter snakes and leopard frogs. Science.

[bib27] Burridge M.J., Simmons L.A. (2003). Exotic ticks introduced into the United States on imported reptiles from 1962 to 2001 and their potential roles in international dissemination of diseases. Vet. Parasitol..

[bib28] Burkett-Cadena N.D., Graham S.P., Hassan H.K., Guyer C., Eubanks M.D., Katholi C.R., Unnasch T.R. (2008). Blood feeding patterns of potential arbovirus vectors of the genus Culex targeting ectothermic hosts. Am. J. Trop. Med. Hyg..

[bib29] Burkett-Cadena N.D., McClure C.J., Estep L.K., Eubanks M.D. (2013). Hosts or habitats: what drives the spatial distribution of mosquitoes?. Ecosphere.

[bib30] Bravo-Barriga D., Parreira R., Maia C., Blanco-Ciudad J., Afonso M.O., Frontera E., Campino L., Pérez-Martín J.E., Serrano Aguilera F.J., Reina D. (2016). First molecular detection of *Leishmania tarentolae*-like DNA in *Sergentomyia minuta* in Spain. Parasitol. Res..

[bib31] Camin J.H. (1948). Mite transmission of a hemorrhagic septicemia in snakes. J. Parasitol..

[bib32] Charleston M., Perkins S. (2003). Lizards, malaria, and jungles in the caribbean. Tangled Trees: Phylogeny, Cospeciation and Coevolution.

[bib33] Chen H., Li J., Zhang J., Guo X., Liu J., He J., Chen J. (2019). Multi-locus characterization and phylogenetic inference of *Leishmania* spp. in snakes from Northwest China. PloS One.

[bib34] Charrel R.N., Izri A., Temmam S., De Lamballerie X., Parola P. (2006). Toscana virus RNA in *Sergentomyia minuta* flies. Emerg. Infect. Dis..

[bib35] Chiang C.L., Reeves W.C. (1962). Statistical estimation of virus infection rates in mosquito vector populations. Am. J. Hyg..

[bib36] Chilton N.B., Bull C.M., Andrews R.H. (1992). Differences in attachment site of the Australian reptile tick *Amblyomma limbatum* (Acari: Ixodidae) on two host species. Int. J. Parasitol..

[bib37] Chitimia-Dobler L., Langguth J., Pfeffer M., Kattner S., Küpper T., Friese D., Dobler G., Guglielmone A.A., Nava S. (2017). Genetic analysis of *Rhipicephalus sanguineus* sensu lato ticks parasites of dogs in Africa north of the Sahara based on mitochondrial DNA sequences. Vet. Parasitol..

[bib38] Colunga‐Salas P., Sánchez‐Montes S., Ochoa‐Ochoa L.M., Grostieta E., Becker I. (2020). Molecular detection of the reptile‐associated *Borrelia* group in *Amblyomma dissimile*, Mexico. Med. Vet. Entomol..

[bib39] Coughlan S., Mulhair P., Sanders M., Schonian G., Cotton J.A., Downing T. (2017). The genome of *Leishmania adleri* from a mammalian host highlights chromosome fission in Sauroleishmania. Sci. Rep..

[bib40] Cotteaux-Lautard C., Leparc-Goffart I., Berenger J.M., Plumet S., Pages F. (2016). Phenology and host preferences *Phlebotomus perniciosus* (Diptera: Phlebotominae) in a focus of Toscana virus (TOSV) in South of France. Acta Trop..

[bib41] Crosby F.L., Wellehan J.F., Pertierra L., Wendland L.D., Lundgren A.M., Barbet A.F., Brown M.B. (2021). Molecular characterization of “*Candidatus* Anaplasma testudinis”: an emerging pathogen in the threatened Florida gopher tortoise (*Gopherus polyphemus*). Ticks Tick Borne Dis.

[bib42] Cupp E.W., Zhang D., Yue X., Cupp M.S., Guyer C., Sprenger T.R., Unnasch T.R. (2004). Identification of reptilian and amphibian blood meals from mosquitoes in an eastern equine encephalomyelitis virus focus in central Alabama. Am. J. Trop. Med. Hyg..

[bib43] Dantas-Torres F., Oliveira-Filho E.F., Soares F.A., Souza B.O., Valença R.B., Sá F.B. (2008). Ticks infesting amphibians and reptiles in Pernambuco, Northeastern Brazil. Rev. Bras. Parasitol. Vet..

[bib44] Dantas-Torres F., Mascarenhas-Junior P.B., Dos Anjos H.R., Dos Santos E.M., Correia J. (2019). Tick infestation on caimans: a casual tick-host association in the Atlantic rainforest biome?. Exp. Appl. Acarol..

[bib45] De Oliveira S.V., Bitencourth K., Borsoi A., de Freitas F., Castelo Branco Coelho G., Amorim M., Gazeta G.S. (2018). Human parasitism and toxicosis by *Ornithodoros rietcorreai* (Acari: Argasidae) in an urban area of Northeastern Brazil. Ticks Tick Borne Dis.

[bib46] Desowitz R. (1991). The Malaria Capers.

[bib47] Di Giovanni F., Wilke A., Beier J., Pombi M., Mendoza-Roldan J., Desneux N., Canale A., Lucchi A., Dantas-Torres F., Otranto D., Benelli G. (2021). Parasitic strategies of arthropods of medical and veterinary importance. Entomol. Gen..

[bib48] Dobson S.J., Barker S.C. (1999). Phylogeny of the hard ticks (Ixodidae) inferred from 18S rRNA indicates that the genus Aponomma is paraphyletic. Mol. Phylogenet. Evol..

[bib49] Dudek K., Skórka P., Sajkowska Z.A., Ekner-Grzyb A., Dudek M., Tryjanowski P. (2016). Distribution pattern and number of ticks on lizards. Ticks Tick Borne Dis.

[bib50] Ebani V.V., Fratini F., Ampola M., Rizzo E., Cerri D., Andreani E. (2008). *Pseudomonas* and *Aeromonas* isolates from domestic reptiles and study of their antimicrobial in vitro sensitivity. Vet. Res. Commun..

[bib51] Ebani V.V. (2017). Domestic reptiles as source of zoonotic bacteria: a mini review. Asian Pac J Trop Med.

[bib52] Estrada-Peña A., de la Fuente J. (2018). The fossil record and the origin of ticks revisited. Exp. Appl. Acarol..

[bib166] Estrada-Peña A., Jongejan F. (1999). Ticks feeding on humans: a review of records on human-biting Ixodoidea with special reference to pathogen transmission. Exp. Appl. Acarol..

[bib53] Fain A. (1962). Les Acariens Mesostigmatiques parasites des serpents. Bull Royal Sci Nat Belg.

[bib54] Fain A., Kutzer E., Fordinal E. (1983). *Entonyssus squamatus* spec. nov. (Acari, Entonyssidae) from the lung of the snake, *Elaphe schrencki* Stejneger, 1925. Int. J. Acarol.

[bib55] Fuantos-Gámez B., Romero-Núñez C., Sheinberg-Waisburd G., Bautista-Gómez L., Yarto-Jaramillo E., Heredia-Cardenas R., Miranda-Contreras L. (2020). Successful treatment of *Ophionyssus natricis* with afoxolaner in two Burmese pythons (*Python molurus bivittatus*). Vet. Dermatol..

[bib56] Fielden L.J., Knolhoff L.M., Villarreal S.M., Ryan P. (2011). Underwater survival in the dog tick *Dermacentor variabilis* (Acari:Ixodidae). J. Insect Physiol..

[bib57] Giannelli A., Dantas-Torres F., Otranto D. (2012). Underwater survival of *Rhipicephalus sanguineus* (Acari: Ixodidae). Exp. Appl. Acarol..

[bib58] Giroud P. (1951). Les rickettsioses en Afrique équatoriale. Bull. World Health Organ..

[bib59] González E., Molina R., Aldea I., Iriso A., Tello A., Jiménez M. (2020). Leishmania sp. detection and blood-feeding behaviour of *Sergentomyia minuta* collected in the human leishmaniasis focus of southwestern Madrid, Spain (2012-2017). Transbound Emerg Dis.

[bib168] Graham S.P., Hassan H.K., Chapman T., White G., Guyer C., Unnasch T.R. (2012). Serosurveillance of eastern equine encephalitis virus in amphibians and reptiles from Alabama, USA. Am. J. Trop. Med. Hyg..

[bib60] Güner E.S., Hashimoto N., Kadosaka T., Imai Y., Masuzawa T. (2003). A novel, fast-growing Borrelia sp. isolated from the hard tick *Hyalomma aegyptium* in Turkey. Microbiology.

[bib167] Gutiérrez-Bugallo G., Piedra L.A., Rodriguez M., Bisset J.A., Lourenço-de-Oliveira R., Weaver S.C., Vasilakis N., Vega-Rúa A. (2019). Vector-borne transmission and evolution of Zika virus. Nat. Ecol. Evol..

[bib61] Hayakawa T., Culleton R., Otani H., Horii T., Tanabe K. (2008). Big bang in the evolution of extant malaria parasites. Mol. Biol. Evol..

[bib169] Heisch R.B. (1958). On *Leishmania adleri* sp. nov. from lacertid lizards (*Latastia* sp.) in Kenya. Ann. Trop. Med. Parasitol..

[bib62] Hoogstraal H., Kohls G.M. (1966). *Argas (Microargas) transversus* banks (New Subgenus) (Ixodoidea, Argasidae), a diminutive parasite of the galapagos giant tortoise: redescription of the holotype male and description of the larva. Ann. Entomol. Soc. Am..

[bib63] Jacinavicius F., Bassini-Silva R., Mendoza-Roldan J.A., Pepato A.R., Ochoa R., Welbourn C., Barros-Battesti D.M. (2018). A checklist of chiggers from Brazil, including new records (Acari: Trombidiformes: Trombiculidae and Leeuwenhoekiidae). ZooKeys.

[bib64] Jacobson E.R. (2007). Infectious Diseases and Pathology of Reptiles: Color Atlas and Text. CRC Press.

[bib65] Jeyaprakash A., Hoy M. (2009). First divergence time estimate of spiders, scorpions, mites and ticks (subphylum: chelicerata) inferred from mitochondrial phylogeny. Exp. Appl. Acarol..

[bib66] Ji W., Wang W., Zhao X., Zai J., Li X. (2020). Cross‐species transmission of the newly identified coronavirus 2019‐nCoV. J. Med. Virol..

[bib67] Johnson-Delaney C.A. (1996). Reptile Zoonoses and Threats to Public Health. Reptile Medicine and Surgery.

[bib68] Kar S., Rodriguez S.E., Akyildiz G., Cajimat M.N., Bircan R., Mears M.C., Keles A.G. (2020). Crimean-Congo hemorrhagic fever virus in tortoises and *Hyalomma aegyptium* ticks in East Thrace, Turkey: potential of a cryptic transmission cycle. Parasites Vectors.

[bib170] Kato H., Uezato H., Sato H., Bhutto A.M., Soomro F.R., Baloch J.H., Iwata H., Hashiguchi Y. (2010). Natural infection of the sand fly Phlebotomus kazeruni by Trypanosoma species in Pakistan. Parasites Vectors.

[bib69] Killick-Kendrick R., Lainson R., Rioux J., Sarjanova V.M. (1986). The taxonomy of *Leishmania*-like parasites of reptiles. Leishmania. Taxonomie et phylogenèse. Applications éco-épidémiologiques.

[bib70] Kim S., Guirgis S., Harris D., Keelan T., Mayer M. (1978). Q fever—New York. Center for Disease Control MMWR.

[bib71] Kočíková B., Majláth I., Víchová B., Maliničová L., Pristaš P., Connors V.A., Majláthová V. (2018). Candidatus Cryptoplasma associated with green lizards and Ixodes ricinus ticks, Slovakia, 2004–2011. Emerg. Infect. Dis..

[bib72] Kuo M.M., Lane R.S., Giclas P.C. (2000). A comparative study of mammalian and reptilian alternative pathway of complement-mediated killing of the Lyme disease spirochete (*Borrelia burgdorferi*). J. Parasitol..

[bib73] Kho K.L., Koh F.X., Tay S.T. (2015). Molecular evidence of potential novel spotted fever group rickettsiae, Anaplasma and Ehrlichia species in Amblyomma ticks parasitizing wild snakes. Parasites Vectors.

[bib74] Klatt S., Simpson L., Maslov D.A., Konthur Z. (2019). *Leishmania tarentolae*: taxonomic classification and its application as a promising biotechnological expression host. PLoS Neglected Trop. Dis..

[bib75] Klompen H., Grimaldi D. (2001). First Mesozoic record of a parasitiform mite: a larval argasid tick in Cretaceous amber (Acari: Ixodida: Argasidae). An Entomol Soc Am.

[bib76] Kwak M.L., Chavatte J.M., Chew K.L., Lee B. (2021). Emergence of the zoonotic tick *dermacentor (indocentor) auratus* supino, 1897 (Acari: Ixodidae) in Singapore. Ticks Tick Borne Dis.

[bib77] Lepetz V., Massot M., Chaine A.S., Clobert J. (2009). Climate warming and the evolution of morphotypes in a reptile. Global Change Biol..

[bib78] Lizaso N.M. (1979). Um novo ácaro da familia Heterozerconidae coletado sobre serpentes brasileiras. Descrição de *Heterozercon elegans* sp. n.(Acarina: Mesostigmata). Mem. Inst. Butantan (Sao Paulo).

[bib79] Lizaso N.M. (1982). Novos gêneros e espécies de ácaros (Mesostigmata, Ixodorhynchidae) ectoparasitas de serpentes. Rev. Bras. Zool..

[bib80] Lozano-Sardaneta Y.N., Salas P.C., Pineda L.S., Montes S.S., Ochoa L.O., Fauser I.B. (2018). *Sauroleishmania*, protozoarios asociados con reptiles: distribución, Vectores y Hospederos. Rev Lat Herpetol.

[bib81] Lupescu I., Baraitareanu S. (2015). Emerging diseases associated with ‘new companion animals’: review in zoonoses transmitted by reptiles. Sci Works Ser C Vet Med.

[bib82] Luz H.R., Faccini J.L. (2013). Parasitismo por carrapatos em Anuros no Brasil: revisão. Vet. Zootec..

[bib83] MacDonald A.J., Hyon D.W., Brewington J.B., O'Connor K.E., Swei A., Briggs C.J. (2017). Lyme disease risk in southern California: abiotic and environmental drivers of *Ixodes pacificus* (Acari: Ixodidae) density and infection prevalence with *Borrelia burgdorferi*. Parasites Vectors.

[bib84] Machado-Ferreira E., Vizzoni V.F., Balsemão-Pires E., Moerbeck L., Gazeta G.S., Piesman J., Voloch C.M., Soares C.A. (2016). *Coxiella* symbionts are widespread into hard ticks. Parasitol. Res..

[bib85] Majláthová V., Majláth I., Derdáková M., Víchová B., Peťko B. (2006). *Borrelia lusitaniae* and green lizards (*Lacerta viridis*), Karst Region, Slovakia. Emerg. Infect. Dis..

[bib86] Majláthová V., Majláth I., Hromada M., Tryjanowski P., Bona M., Antczak M., Víchová B., Dzimko Š., Mihalca A., Peťko B. (2008). The role of the sand lizard (*Lacerta agilis*) in the transmission cycle of Borrelia burgdorferi sensu lato. *I*nt J Med Microbiol.

[bib171] Maleki-Ravasan N., Javadian E., Mohebali M., Dalimi Asl A., Sadraei J., Zarei Z.A., Oshaghi M.A. (2008). Natural infection of sand flies *Sergentomyia dentata* in Ardebil to Lizard Leishmania. Pathobiology.

[bib87] Mans B., de Castro M., Pienaar R., de Klerk D., Gaven P., Genu S., Latif A. (2016). Ancestral reconstruction of tick lineages. Ticks Tick Borne Dis.

[bib88] Manson-Bahr P.E.C., Heisch R.B. (1961). Transient infection of man with a *Leishmania* (*L. adleri*) of lizards. Ann. Trop. Med. Parasitol..

[bib89] Martins T.F., Teixeira R.H., Benatti R., Minervino A.H., Soares H.S., Soares J.F., Labruna M.B. (2020). Life cycle of the tick *Amblyomma humerale* (Parasitiformes: Ixodida) in the laboratory. Int. J. Acarol.

[bib90] Mendoza-Roldan J.A., Bassini-Silva R., Jacinavicius F.C., Nieri-Bastos F.A., Franco F.L., Marcili A., Barros-Battesti D.M. (2017). A new species of pit mite (Trombidiformes: harpirhynchidae) from the South American rattlesnake (Viperidae): morphological and molecular analysis. Entomol., Ornithol. Herpetol..

[bib91] Mendoza-Roldan J.A., Colella V., Lia R.P., Nguyen V.L., Barros-Battesti D.M., Iatta R., Dantas-Torres F., Otranto D. (2019). *Borrelia burgdorferi* (sensu lato) in ectoparasites and reptiles in southern Italy. Parasites Vectors.

[bib92] Mendoza-Roldan J., Ribeiro S.R., Castilho-Onofrio V., Grazziotin F.G., Rocha B., Ferreto-Fiorillo B., Pereira J.S., Benelli G., Otranto D., Barros-Battesti D.M. (2020). Mites and ticks of reptiles and amphibians in Brazil. Acta Trop..

[bib93] Mendoza-Roldan J., Modry D., Otranto D. (2020). Zoonotic parasites of reptiles: a crawling threat. Trends Parasitol..

[bib94] Mendoza-Roldan J., Ribeiro S.R., Castilho-Onofrio V., Marcili A., Simonato B.B., Latrofa M.S., Benelli G., Otranto D., Barros-Battesti D.M. (2021). Molecular detection of vector-borne agents in ectoparasites and reptiles from Brazil. Ticks Tick Borne Dis.

[bib95] Mendoza-Roldan J.A., Ravindran Santhakumari Manoj R., Latrofa M.S., Iatta R., Annoscia G., Lovreglio P., Stufano A., Dantas-Torres F., Davoust B., Laidoudi Y., Mediannikov O., Otranto D. (2021). Role of reptiles and associated arthropods in the epidemiology of rickettsioses: a one health paradigm. PLoS Neglected Trop. Dis..

[bib96] Miranda R.J., Cleghorn J.E., Bermudez S.E., Perotti M.A. (2017). Occurrence of the mite *Ophionyssus natricis* (Acari: Macronyssidae) on captive snakes from Panama. Acarologia.

[bib97] Mihalca A.D. (2015). Ticks imported to Europe with exotic reptiles. Vet. Parasitol..

[bib98] Morales-Diaz J., Colunga-Salas P., Romero-Salas D., Sánchez-Montes S., Estrada-Souza I.M., Ochoa-Ochoa L.M., Cruz-Romero A. (2020). Molecular detection of reptile-associated *Borrelia* in *Boa constrictor* (Squamata: boidae) from veracruz, Mexico. Acta Trop..

[bib99] Muñoz-Leal S., Toledo L.F., Venzal J.M., Marcili A., Martins T.F., Acosta I., Pinter A., Labruna M.B. (2017). Description of a new soft tick species (Acari: Argasidae: Ornithodoros) associated with stream-breeding frogs (Anura: cycloramphidae: *Cycloramphus*) in Brazil. Ticks Tick Borne Dis.

[bib100] Njagu Z., Mihok S., Kokwaro E., Verloo D. (1999). Isolation of *Trypanosoma brucei* from the monitor lizard (*Varanus niloticus*) in an endemic focus of Rhodesian sleeping sickness in Kenya. Acta Trop..

[bib101] Nieto N.C., Foley J.E., Bettaso J., Lane R.S. (2009). Reptile infection with *Anaplasma phagocytophilum*, the causative agent of granulocytic anaplasmosis. J. Parasitol..

[bib102] Nowak M., Cieniuch S., Stańczak J., Siuda K. (2010). Detection of *Anaplasma phagocytophilum* in *Amblyomma flavomaculatum* ticks (Acari: Ixodidae) collected from lizard *Varanus exanthematicus* imported to Poland. Exp. Appl. Acarol..

[bib103] Norval G., Sharrad R.D., Gardner M.G. (2020). Three instances of reptile ticks parasitising humans. Acarologia.

[bib104] Novo S.P., Leles D., Bianucci R., Araujo A. (2015). *Leishmania tarentolae* molecular signatures in a 300 hundred-years-old human Brazilian mummy. Parasites Vectors.

[bib105] Novakova M., Literak I., Chevez L., Martins T.F., Ogrzewalska M., Labruna M.B. (2015). Rickettsial infections in ticks from reptiles, birds and humans in Honduras. Ticks Tick Borne Dis.

[bib106] Ogrzewalska M., Pinter A. (2016). Ticks (Acari: Ixodidae) as ectoparasites of Brazilian wild birds and their association with rickettsial diseases. Braz. J. Vet. Res. Anim. Sci..

[bib107] Ogrzewalska M., Machado C., Rozental T., Forneas D., Cunha L.E., De Lemos E.R.S. (2019). Microorganisms in the ticks *Amblyomma dissimile* Koch 1844 and *Amblyomma rotundatum* Koch 1844 collected from snakes in Brazil. Med. Vet. Entomol..

[bib108] Omondi D., Masiga D.K., Fielding B.C., Kariuki E., Ajamma Y.U., Mwamuye M.M., Villinger J. (2017). Molecular detection of tick-borne pathogen diversities in ticks from livestock and reptiles along the shores and adjacent islands of Lake Victoria and Lake Baringo, Kenya. Front Vet Sci.

[bib109] Oya A., Shirasaka A., Yabe S., Sasa M. (1983). Studies on Japanese encephalitis virus infection of reptiles. I. Experimental infection of snakes and lizards. Jpn. J. Exp. Med..

[bib110] Ovezmukhammedov A. (1991). Leishmania of Reptiles. Leishmania of Reptiles.

[bib111] Panetta J.L., Šíma R., Calvani N., Hajdušek O., Chandra S., Panuccio J., Šlapeta J. (2017). Reptile-associated *Borrelia* species in the goanna tick (*Bothriocroton undatum*) from Sydney, Australia. Parasites Vectors.

[bib112] Parola P., Vestris G., Martinez D., Brochier B., Roux Raoult D. (1999). Tick-borne rickettiosis in Guadeloupe, the French West Indies: isolation of *Rickettsia africae* from *Amblyomma variegatum* ticks and serosurvey in humans, cattle, and goats. Am. J. Trop. Med. Hyg..

[bib113] Parola P., Paddock C.D., Raoult D. (2005). Tick-borne rickettsioses around the world: emerging diseases challenging old concepts. Clin. Microbiol. Rev..

[bib114] Paștiu A.I., Matei I.A., Mihalca A.D., D'Amico G., Dumitrache M.O., Kalmár Z., Sándor A.D., Lefkaditis M., Gherman C.M., Cozma V. (2012). Zoonotic pathogens associated with *Hyalomma aegyptium* in endangered tortoises: evidence for host-switching behaviour in ticks?. Parasites Vectors.

[bib115] Peñalver E., Arillo A., Delclòs X., Peris D., Grimaldi D.A., Anderson S.R., Nascimbene P.C., Pérez-de la Fuente R. (2017). Ticks parasitised feathered dinosaurs as revealed by Cretaceous amber assemblages. Nat. Commun..

[bib116] Pérez-Cutillas P., Muñoz C., Martínez-De La Puente J., Figuerola J., Navarro R., Ortuño M., Bernal L.J., Ortiz J., Soriguer R.C., Berriatua E. (2020). A spatial ecology study in a high-diversity host community to understand blood-feeding behaviour in *Phlebotomus* sandfly vectors of *Leishmania*. Med. Vet. Entomol..

[bib117] Peter T.F., Burridge M.J., Mahan S.M. (2002). *Ehrlichia ruminantium* infection (heartwater) in wild animals. Trends Parasitol..

[bib118] Pombi M., Giacomi A., Barlozzari G., Mendoza‐Roldan J., Macrì G., Otranto D., Gabrielli S. (2020). Molecular detection of *Leishmania (Sauroleishmania) tarentolae* in human blood and *Leishmania (Leishmania) infantum* in *Sergentomyia minuta*: unexpected host‐parasite contacts. Med. Vet. Entomol..

[bib119] Pincheira-Donoso D., Bauer A.M., Meiri S., Uetz P. (2013). Global taxonomic diversity of living reptiles. PloS One.

[bib120] Pietzsch M., Quest R., Hillyard P.D., Medlock J.M., Leach S. (2006). Importation of exotic ticks into the United Kingdom via the international trade in reptiles. Exp. Appl. Acarol..

[bib121] Polo G., Luz H.R., Regolin A.L., Martins T.F., Winck G.R., da Silva H.R., Faccini J.L. (2021). Distribution modeling of *Amblyomma rotundatum* and *Amblyomma dissimile* in Brazil: estimates of environmental suitability. Parasitol. Res..

[bib122] Poinar G., Brown A.E. (2003). A new genus of hard ticks in Cretaceous Burmese amber (Acari: Ixodida: Ixodidae). Sys parasitol.

[bib123] Poinar G., Poinar R. (2004). Evidence of vector-borne disease of Early Cretaceous reptiles. Vector Borne Zoonotic Dis..

[bib124] Poinar G., Poinar R. (2004). *Paleoleishmania proterus* n. gen., n. sp., (Trypanosomatidae: kinetoplastida) from Cretaceous Burmese amber. Protist.

[bib125] Poinar G. (2008). *Lutzomyia adiketis* sp. n.(Diptera: Phlebotomidae), a vector of *Paleoleishmania neotropicum* sp. n.(Kinetoplastida: Trypanosomatidae) in Dominican amber. Parasites Vectors.

[bib126] Prakasan K., Aiswarya M., Aswathi R. (2020). Climate-the principal factor influencing tick plethora and tick-borne zoonoses: a Review. Uttar Pradesh J. Zool..

[bib127] Prendeville H.R., Hanley K.A. (2000). Prevalence of the tick, *Ixodes pacificus*, on western fence lizards, *Sceloporus occidentalis*: trends by gender, size, season, site, and mite infestation. J. Herpetol..

[bib128] Qiu Y., Kidera N., Hayashi M., Fujishima K., Tamura H. (2021). Rickettsia spp. and Ehrlichia spp. in Amblyomma ticks parasitizing wild amphibious sea kraits and yellow-margined box turtles in Okinawa, Japan. Ticks Tick Borne Dis.

[bib129] Ready P.D. (2013). Biology of phlebotomine sand flies as vectors of disease agents. Annu. Rev. Entomol..

[bib130] Reeves W.K. (2009). *Lutzomyia (helcocyrtomyia) Apache* young and Perkins (Diptera: Psychodidae) feeds on reptiles. Entomol. News.

[bib131] Rezende J., Rangel C.P., Cunha N.C., Fonseca A.H. (2012). Primary embryonic cells of *Rhipicephalus microplus* and *Amblyomma cajennense* ticks as a substrate for the development of *Borrelia burgdorferi* (strain G39/40). Braz. J. Biol..

[bib132] Rissmann M., Kley N., Ulrich R., Stoek F., Balkema-Buschmann A., Eiden M., Groschup M.H. (2020). Competency of amphibians and reptiles and their potential role as reservoir hosts for Rift Valley Fever Virus. Viruses.

[bib133] Roll U., Feldman A., Novosolov M., Allison A., Bauer A.M., Bernard R., Böhm M., Castro-Herrera F., Chirio L., Collen B., Colli G.R., Dabool L., Das I., Doan T.M., Grismer L.L., Hoogmoed M., Itescu Y., Kraus F., LeBreton M., Lewin A., Martins M., Maza E., Meirte D., Nagy Z.T., de C Nogueira C., Pauwels O.S.G., Pincheira-Donoso D., Powney G.D., Sindaco R., Tallowin O.J.S., Torres-Carvajal O., Trape J.F., Vidan E., Uetz P., Wagner P., Wang Y., Orme C.D.L., Grenyer R., Meiri S. (2017). The global distribution of tetrapods reveals a need for targeted reptile conservation. Nat Ecol Evol.

[bib134] Sánchez-Montes S., Isaak-Delgado A.B., Guzmán-Cornejo C., Rendón-Franco E., Muñoz-García C.I., Bermúdez S., Becker I. (2019). *Rickettsia* species in ticks that parasitize amphibians and reptiles: novel report from Mexico and review of the worldwide record. Ticks Tick Borne Dis.

[bib165] Santodomingo A., Cotes-Perdomo A., Foley J., Castro L.R. (2018). Rickettsial infection in ticks (Acari: Ixodidae) from reptiles in the Colombian Caribbean. Ticks Tick Borne Dis..

[bib135] Schultz H. (1975). Human infestation by *Ophionyssus natricis* snake mite. Br. J. Dermatol..

[bib136] Soualah-Alila H., Bouslama Z., Amr Z., Bani Hani R. (2015). Tick infestations (Acari: Ixodidae) on three lizard species from Seraidi (Annaba District), northeastern Algeria. Exp. Appl. Acarol..

[bib137] Shahhosseini N., Friedrich J., Moosa-Kazemi S.H., Sedaghat M.M., Kayedi M.H., Tannich E., Schmidt-Chanasit J., Lühken R. (2018). Host-feeding patterns of *Culex* mosquitoes in Iran. Parasites Vectors.

[bib138] Steinman A., Banet-Noach C., Tal S., Levi O., Simanov L., Perk S., Shpigel N. (2003). West Nile virus infection in crocodiles. Emerg. Infect. Dis..

[bib139] Stenos J., Graves S., Popov V.L., Walker D.H. (2003). *Aponomma hydrosauri*, the reptile-associated tick reservoir of *Rickettsia honei* on Flinders Island, Australia. Am. J. Trop. Med. Hyg..

[bib140] Špitalská E., Sparagano O., Stanko M., Schwarzová K., Špitalský Z., Škultéty Ľ., Havlíková S.F. (2018). Diversity of *Coxiella*-like and *Francisella*-like endosymbionts, and *Rickettsia* spp., *Coxiella burnetii* as pathogens in the tick populations of Slovakia, Central Europe. Ticks Tick Borne Dis.

[bib141] Široký P., Kubelová M., Modrý D., Erhart J., Literák I., Špitalská E., Kocianová E. (2010). Tortoise tick *Hyalomma aegyptium* as long-term carrier of Q fever agent *Coxiella burnetii*—evidence from experimental infection. Parasitol. Res..

[bib142] Široký P., Bělohlávek T., Papoušek I., Jandzik D., Mikulíček P., Kubelová M., Zdražilová-Dubská L. (2014). Hidden threat of tortoise ticks: high prevalence of Crimean-Congo haemorrhagic fever virus in ticks *Hyalomma aegyptium* in the Middle East. Parasites Vectors.

[bib143] Silva-Santos C., Pie M.R., da Rocha T.C., Navarro-Silva M.A. (2019). Molecular identification of blood meals in mosquitoes (Diptera, Culicidae) in urban and forested habitats in southern Brazil. PloS One.

[bib144] Szekeres S., Majláthová V., Majláth I., Földvári G. (2016). Neglected hosts: the role of lacertid lizards and medium-sized mammals in the eco-epidemiology of Lyme borreliosis. Ecology and Prevention of Lyme Borreliosis.

[bib145] Takano A., Goka K., Une Y., Shimada Y., Fujita H., Shiino T., Kawabata H. (2010). Isolation and characterization of a novel *Borrelia* group of tick‐borne borreliae from imported reptiles and their associated ticks. Environ. Microbiol..

[bib146] Takano A., Kuwata R., Shimoda H., Hadi U.K., Setiyono A., Agungpriyono S., Maeda K. (2019). Detection and isolation of tick‐borne bacteria (*Anaplasma* spp., *Rickettsia* spp., and *Borrelia* spp.) in *Amblyomma varanense* ticks on lizard (*Varanus salvator*). Microbiol. Immunol..

[bib147] Telford S.R. (2009). Hemoparasites of the Reptilia. CRC Press.

[bib148] Tijsse-Klasen E., Fonville M., Reimerink J.H., Spitzen-van der Sluijs A., Sprong H. (2010). Role of sand lizards in the ecology of Lyme and other tick-borne diseases in The Netherlands. Parasites Vectors.

[bib149] Tiwari R., Dhama K., Sharun K., Iqbal Yatoo M., Malik Y.S., Singh R., Rodriguez-Morales A.J. (2020). COVID-19: animals, veterinary and zoonotic links. Vet. Q..

[bib150] Thomas L.A., Eklund C.M. (1962). Overwintering of western equine encephalomyelitis virus in garter snakes experimentally infected by *Culex tarsalis*. Proc Soc Exp Biol Med.

[bib151] Thomas S.G., Glover M.A., Parthasarathy A., Wong N.H., Shipman P.A., Hudson A.O. (2020). Expression of a shiga-like toxin during plastic colonization by two multidrug-resistant bacteria, *Aeromonas hydrophila* RIT668 and *Citrobacter freundii* RIT669, isolated from endangered turtles (*Clemmys guttata*). Microorganisms.

[bib152] Torres-Guerrero E., Quintanilla-Cedillo M.R., Ruiz-Esmenjaud J., Arenas R. (2017). Leishmaniasis: a review. F1000Research.

[bib153] Tucker E., Benton J. (1982). Triassic environments, climates and reptile evolution. Palaeogeogr. Palaeoclimatol. Palaeoecol..

[bib154] Tuon F.F., Amato Neto V., Sabbaga Amato V. (2008). *Leishmania*: origin, evolution and future since the Precambrian. EMS Immunol Med Microbiol.

[bib155] Václav R., Ficová M., Prokop P., Betáková T. (2011). Associations between coinfection prevalence of *Borrelia lusitaniae, Anaplasma* sp., and *Rickettsia* sp. in hard ticks feeding on reptile hosts. Microb. Ecol..

[bib156] Valentine K.H., Harms C.A., Cadenas M.B., Birkenheuer A.J., Marr H.S., Braun-McNeill J., Breitschwerdt E.B. (2007). *Bartonella* DNA in loggerhead sea turtles. Emerg. Infect. Dis..

[bib157] Viola L.B., Campaner M., Takata C.S., Ferreira R.C., Rodrigues A.C., Freitas R.A.D., Teixeira M.M.G. (2008). Phylogeny of snake trypanosomes inferred by SSU rDNA sequences, their possible transmission by phlebotomines, and taxonomic appraisal by molecular, cross-infection and morphological analysis. Parasitology.

[bib158] Waiswa C., Picozzi K., Olaho‐Mukani W., Katunguka‐Rwakishaya E. (2003). Monitor lizard (*Varanus niloticus*, Linnaeus, 1766) as a host for tsetse (*Glossina fuscipes fuscipes*, Newstead, 1910) in the sleeping sickness endemic foci of Uganda. Afr. J. Ecol..

[bib159] Weinert L.A., Werren J.H., Aebi A., Stone G.N., Jiggins F.M. (2009). Evolution and diversity of *Rickettsia* bacteria. BMC Biol..

[bib160] Whiley H., Custance G., Graves S., Stenos J., Taylor M., Ross K., Gardner M.G. (2016). *Rickettsia* detected in the reptile tick *Bothriocroton hydrosauri* from the lizard *Tiliqua rugosa* in South Australia. Pathogens.

[bib161] Wieczorek M., Rektor R., Najbar B., Morelli F. (2020). Tick parasitism is associated with home range area in the sand lizard, *Lacerta agilis*. Amphibia-Reptilia.

[bib162] Woo P., Soltys M.A. (1969). The experimental infection of reptiles with *Trypanosoma brucei*. Ann. Trop. Med. Parasitol..

[bib163] Zhang L., Zhang Y., Adusumilli S., Liu L., Narasimhan S., Dai J., Zhao Y.O., Fikrig E. (2011). Molecular interactions that enable movement of the Lyme disease agent from the tick gut into the hemolymph. PLoS Pathog..

[bib164] Zhang J.R., Guo X.G., Chen H., Liu J.L., Gong X., Chen D.L., Chen J.P. (2019). Pathogenic *Leishmania* spp. detected in lizards from Northwest China using molecular methods. BMC Vet. Res..

